# Emotion Processing in Peripheral Neuropathic Pain: An Observational Study

**DOI:** 10.3390/medsci12020027

**Published:** 2024-05-17

**Authors:** Gianluca Isoardo, Mauro Adenzato, Stefano Ciullo, Elena Fontana, Ilaria Stura, Giuseppe Migliaretti, Paolo Titolo, Enrico Matteoni, Andrea Calvo, Federica Laino, Francesca Palumbo, Rita B. Ardito

**Affiliations:** 1 Department of Neurosciences & Mental Health, Hospital “Città della Salute e della Scienza di Torino”, 10126 Turin, Italy; gianlucaisoardo@gmail.com; 2 Department of Psychology, University of Turin, 10124 Turin, Italy; mauro.adenzato@unito.it (M.A.); stefanociullo@gmail.com (S.C.); elena.fo@live.it (E.F.); 3 Department of Neuroscience “Rita Levi Montalcini”, University of Turin, 10126 Turin, Italy; ilariastura@gmail.com (I.S.); enricomatteoni12@gmail.com (E.M.); andrea.calvo@unito.it (A.C.); francesca.palumbo@unito.it (F.P.); 4 Department of Public Health and Pediatric Sciences, University of Turin, 10126 Torino, Italy; giuseppe.migliaretti@unito.it; 5 UOD Reconstructive Microsurgery, Department of Orthopedics & Traumatology, Hospital “Città della Salute e della Scienza di Torino”, 10126 Turin, Italy; titolopaolo@gmail.com; 6 1st Neurology Unit, Department of Neurosciences & Mental Health, Hospital “Città della Salute e della Scienza di Torino”, 10126 Turin, Italy; 7 Unit of Pain Management and Palliative Care, Department of Anesthesia, Intensive Care and Emergency, Hospital “Città della Salute e della Scienza di Torino”, 10126 Turin, Italy; flaino@cittadellasalute.to.it

**Keywords:** alexithymia, emotion, nerve conduction studies, neuropathic pain, quantitative sensory testing, somatosensory functions

## Abstract

Background: In clinical practice, the implementation of tailored treatment is crucial for assessing the patient’s emotional processing profile. Here, we investigate all three levels of analysis characterizing emotion processing, i.e., recognition, representation, and regulation, in patients with peripheral neuropathic pain (PNP). Methods: Sixty-two patients and forty-eight healthy controls underwent quantitative sensory testing, i.e., psychophysical tests to assess somatosensory functions such as perception of cold (CDT), heat-induced pain (HPT), and vibration (VDT), as well as three standardized tasks to assess emotional processing: (1) the Ekman 60-Faces Test (EK-60F) to assess recognition of basic facial emotions, (2) the Reading the Mind in the Eyes Test (RME) to assess the ability to represent the feelings of another person by observing their eyes, and (3) the 20-item Toronto Alexithymia Scale (TAS-20) to assess emotional dysregulation, i.e., alexithymia. Results: General Linear Model analysis revealed a significant relationship between left index finger VDT z-scores in PNP patients with alexithymia. The RME correlated with VDT z-scores of the left little finger and overall score for the EK-60F. Conclusions: In patients with PNP, emotion processing is impaired, which emphasizes the importance of assessing these abilities appropriately in these patients. In this way, clinicians can tailor treatment to the needs of individual patients.

## 1. Introduction

In 2020, the International Association for the Study of Pain (IASP) defined pain as “an unpleasant sensory and emotional experience associated with, or resembling that associated with, actual or potential tissue damage” [1]. This definition emphasized the sensory and affective dimensions of the pain experience, with the latter being prominent in chronic primary pain [2]. Emotions have been variously defined since the seminal work of Charles Darwin and William James [3,4]. In Rolls’ view [5], emotions are states that are associated with rewards and punishments and drive actions to obtain the reward and avoid the punishment. In this model, pain was included among the primary means of punishment that reduce the probability of approach behavior. Malezieux and colleagues [4] defined emotions as functional states and recognized that pain, along with other physiological need states, shares some characteristics of emotions, such as valence, global coordination and pleiotropy, intensity, priority, generalization, and persistence.

Regardless of whether one considers pain as a punisher that triggers emotions or a functional state per se, the link between emotion regulation and pain is further highlighted by the role of alexithymia in various types of pain, including chronic primary pain such as fibromyalgia [6] and neuropathic pain [7]. Alexithymia is a personality construct characterized by an impairment in identifying feelings and associating them with bodily sensations of emotional arousal, an inability to find appropriate words to describe feelings, and a preoccupation with external details of events [8,9,10].

One of the main problems in the investigation of emotional deficits in neuropathic pain is an atomizing approach that does not consider the different levels of emotion processing. Following previous studies in the field of clinical neuroscience [6,11,12], in this paper, we present an analysis that simultaneously considers the three different levels that characterize emotion processing: (1) the identification of a specific emotion (emotion recognition), (2) the attribution of one specific affective mental state to another (emotion representation), and (3) the management of one’s own emotion (emotion regulation), i.e., the ability that is impaired in people with alexithymia. Accordingly, here we used three standardized emotional tasks to test emotional processing in people with peripheral neuropathic pain (PNP): (1) the Ekman 60-Faces Test (EK-60F) to assess the recognition of basic facial emotions, (2) the Reading the Mind in the Eyes Test (RME) to assess the ability to represent the feelings of another person by observing only their eyes, and (3) the 20-item Toronto Alexithymia Scale (TAS-20) to assess the three facets of emotion regulation, namely difficulty identifying feelings, difficulty describing feelings, and externally oriented thinking.

In a previous study [7], we found higher alexithymia in patients with PNP than in healthy controls and we found a relationship between the overall score of the TAS-20 and the TAS-20 subscore for difficulty identifying feelings and the left index vibratory perception threshold (VDT) assessed by quantitative sensory testing (QST). QST is a reliable and reproducible tool for detecting allodynia and hypoesthesia in a neuroanatomically plausible distribution, which is a crucial step in the grading system of certainty for the diagnosis of neuropathic pain [13,14,15,16]. Furthermore, QST allows the definition of the association of abnormalities of different sensory modalities, i.e., the sensory phenotype, which can indicate a possible underlying pathophysiology of pain [17,18] and predict treatment outcome [19,20,21].

The aim of this study is to extend the observations of a previous study [7] by considering and evaluating all three levels of analysis that characterize emotion processing, i.e., emotion recognition, emotion representation and emotion regulation, in patients with PNP within a unified theoretical and methodological framework. We also wanted to investigate the correlation between the QST parameters and emotion processing.

## 2. Materials and Methods

### 2.1. Participants

The ethics committee of the Hospital “Città della Salute e della Scienza” in Turin, Italy, approved this study (protocol number 2CEI778). All patients and control participants gave their informed consent to participate in the study. Forty-eight healthy controls and sixty-two patients with PNP in at least one hand were recruited. We decided to recruit only patients with PNP in the hands because the skin of the hand is densely innervated with specialized cutaneous afferents that project to the somatosensory cortex [22] and because these projections contribute to the experience of emotions [23].

Inclusion criteria were chronic (of more than 3 months duration) PNP [13,14,24] and diagnosis of carpal tunnel syndrome (CTS), brachial plexopathy, painful cervical radiculopathy, ulnar neuropathy at the elbow, or hypertrophic post-burn scars (PBHS). The diagnosis of CTS was made according to the criteria of the American Academy of Neurology [25] and the American Association of Electrodiagnostic Medicine [26]. Brachial plexopathy and painful radiculopathy [14] were diagnosed on the basis of neurophysiological [27] and/or MRI findings. Ulnar neuropathy at the elbow was diagnosed according to [28] and PBHS according to [29]. Participants were excluded if they were younger than 14 years or older than 80 years, could not perform the QST assessment, had a history of alcohol or drug abuse, or were diagnosed with hereditary or acquired central nervous system disorders or polyneuropathy. Data from patients with brachial plexopathy, painful cervical radiculopathy, and ulnar neuropathy were considered together, as previously described [7]. All patients underwent a full clinical examination according to [7], including assessment of pinprick, touch, and position sense in both upper limbs. In addition to the assessing pinprick and touch, pain sites were also assessed for signs of allodynia in response to brushing using the Douleur Neuropathique 4-question (DN4) questionnaire [30]. Pain intensity in the 7 days prior to the evaluation was defined using the Numerical Rating Scale (NRS) [31] as described in [7].

### 2.2. Nerve Conduction Studies (NCSs)

Participants underwent bilateral motor NCS of the median and ulnar nerves and antidromic sensory NCS of the median, radial, and ulnar nerves according to the standard techniques described previously [7].

### 2.3. Quantitative Sensory Testing (QST)

The QST was performed as previously described [7]. The thresholds for the perception of cold (CDT), heat-induced pain (HPT), and vibration (VDT) were evaluated on the palmar surface of the index and little fingers. CDT and HPT were also evaluated on the dorsum of the hand. VDT, CDT, and HPT were assessed using a commercially available thermal and vibratory stimulation device (Medoc TSA II, Durham, NC, USA). HPT was assessed using the method of limits, CDT was assessed using the staircase method with null stimulations, and VDT was assessed using the method of levels with null stimulations.

We defined hypoesthesia as follows: CDT z-scores less than −2.58 (cold hypoesthesia), VDT z-score greater than 2.58 (vibration hypoesthesia), and no pain perception at 50 °C (heat pain hypoesthesia). We defined allodynia for heat pain when the HPT z-score was lower than −1.64. We defined the sensory phenotype based on the QST and DN4 questionnaire results. According to [32], thermal or pain (either heat or pinprick) hypoesthesia was labeled as L1, vibration or touch hypoesthesia as L2, the presence of both thermal/pain and vibration/touch hypoesthesia as L3, and no sensory impairment as L0. Allodynia to heat was labeled as G1, allodynia to mechanical stimulation as G2, combined allodynia to heat and mechanical stimuli as G3, and no allodynia as G0.

### 2.4. Emotion Processing and General Health Assessment

Emotion recognition was assessed by means of the validated Ekman 60-Faces Test (EK-60F). This is a task in which photographs of the faces of 10 actors selected by [33] are used to measure the ability to recognize facial expressions associated with the following six basic emotions: anger, disgust, fear, happiness, sadness, surprise. The images were presented on the computer in a pseudorandom order. Each participant had to verbally indicate which of the six labels for the basic emotions listed under each picture best described the facial expression shown. A maximum score of 60 points could be achieved in this test, i.e., 10 points for each of the six basic emotions.

Emotion representation was assessed using the validated Reading the Mind in the Eyes Test (RME) [34]. This is a task that is frequently used to assess the affective Theory of Mind (ToM), i.e., the ability to infer the emotions and feelings of other people [35,36]. The RME consists of the presentation of 36 black and white photos of the eye region of 36 different human faces. Each participant was shown the photographs on a computer screen and asked to choose which of the four words printed below the photo best describes what the person in the photo is thinking or feeling. Participants give their answers verbally. One point is awarded for each correct answer, with the maximum number of points being 36.

Emotion regulation was assessed by means of the validated 20-item Toronto Alexithymia Scale (TAS-20) [37,38], in which the 20 items are rated on a 5-point Likert scale. The results provide a TAS-20 total score based on the outcome of three subscales that assess different aspects of alexithymia: difficulty identifying feelings, difficulty describing feelings, and externally oriented thinking. The TAS-20 cut-off scores are: ≤51 no alexithymia, 52–60 borderline (or moderate) alexithymia, and ≥61 (high) alexithymia.

To assess levels of psychological distress, we also administered the 12-item General Health Questionnaire (GHQ-12) [39]. The GHQ-12 is a self-report in which the items are rated on a 1–4 Likert scale. Higher scores indicate a higher level of psychological distress, with a cut-off value of ≥4.

## 3. Statistical Analysis

Data are presented as mean ± standard deviation (SD) for continuous variables and as absolute and relative frequencies for categorical variables. The normality of the distribution of the quantitative parameters was tested by means of the Kolmogorov–Smirnov test, and QST parameters that were not normally distributed were log-transformed to be analyzed using parametric methods of inferential analysis [7,40]. The CDT, HPT, and VDT z-scores of patients at each site were calculated as follows:

(log/ln patient value − mean log/ln healthy controls values)/SD log/ln healthy controls values

To avoid overestimating statistical significance when comparing patient and control hands due to the inclusion of patients with bilateral pain, a further comparison by patient was performed [41]. Differences between groups of hands/patients (with painful hands, with non-painful hands and healthy hands) were analyzed using a Mann–Whitney or Wilcoxon test. Categorical data were compared using a Chi-square test or Fisher’s exact test where appropriate. Correlations were analyzed by estimating the parametric r-Pearson correlation coefficient. General Linear Models (GLMs) were used to test the dependence of VDT on the other parameters. Group, class, and educational age were considered as possible confounding factors; if significant, the analyses are presented subdivided according to these variables. Statistical analyses were performed using SAS^®^ Statistics Software 9.4. In all analyses, *p*-values < 0.05 were considered statistically significant. The sample size was set to detect at least a 10% difference in TAS-20 total score between patients and controls, achieving a power of 80% with a two-tailed alpha error of 0.05, as reported in our previous study [7].

## 4. Results

### 4.1. Demographic, Clinical, and NCS Findings

As psychological characteristics and quality of health were correlated with the presence of alexithymia in our previous study [7] but not with the diagnosis, patients were categorized as moderate/high alexithymic (TAS-20 ≥ 52) and non-alexithymic (TAS-20 < 52) in subsequent analyses. The demographic and clinical characteristics of all patients, non-alexithymic and moderate/high alexithymic patients, and healthy controls are summarized in Table 1.

The educational qualifications of the patients and the control group differed significantly in terms of the number of years of education and the distribution of subjects, with the patient group more frequently having a primary and secondary school qualification, while the control group more frequently had a university degree. In 30 patients, the hands were affected bilaterally by neuropathic pain, in 16 patients the right hand was affected, and in 16 patients the left hand was affected. Mean NRS and DN4 also did not differ between non-alexithymic and moderate/high alexithymic patients (Table 1). The NCS findings in patients and healthy controls are summarized in Supplementary Table S1.

### 4.2. QST Evaluation and Sensory Phenotype

The QST results are summarized in Table 2 (z-scores) and in Supplementary Table S2 (log and ln-transformed data). The log-transformed CDT values were significantly lower in patients than in healthy controls on the left dorsum, left little finger, and bilateral index finger, and ln-transformed VDT values were higher on the bilateral index and little finger of patients than in healthy controls. No significant differences were found between the sides for the log-transformed CDT and HPT values and the ln-transformed VDT values in either patients or controls. Left index CDT and VDT z-scores were higher in alexithymic patients than in non-alexithymic patients.

### 4.3. Emotion Processing and Health Quality Evaluation

The results of the EK-60F overall score and its subscores, RME, and GHQ-12 are summarized in Table 3. The overall score of the EK-60F was lower in patients than in healthy controls (46.9 ± 5.7 vs. 49.1 ± 5.1, *p* = 0.04). No differences were found between patients and controls or between CTS, ONP, and PBHS and the EK-60F subscores and RME. Alexithymic patients had a lower overall EK-60F and RME score and a higher GHQ-12 than controls, a lower EK-60F subscore for anger than controls and non-alexithymic patients, and a lower EK-60F subscore for sadness than non-alexithymic patients.

### 4.4. Correlation Analysis between Emotion Processing Characteristics

In the patients, the RME correlated with the overall score of the EK-60F (r = 0.57, *p* < 0.0001) and the subscores for surprise (r = 0.47, *p* = 0.0002), anger (r = 0.32, *p* = 0.01), and fear (r = 0.4, *p* = 0.0003). The overall score of the EK-60F inversely correlated with the TAS-20 overall score (r = −0.28, *p* = 0.03). In non-alexithymic patients, the RME correlated with the overall score of the EK-60F (r = 0.64, *p* < 0.0001) and the subscores for surprise (r = 0.47, *p* = 0.003), anger (r = 0.42, *p* = 0.01), sadness (r = 0.34, *p* = 0.03), and fear (r = 0.55, *p* = 0.0005). In contrast, in the alexithymic patients, the RME correlated only with the EK-60F subscore for surprise (r = 0.53, *p* = 0.01).

### 4.5. Correlation Analysis between QST Profile and Emotion Processing Characteristics

In the patients, the VDT z-score at the left index finger inversely correlated with the RME (r = −0.33, *p* = 0.02) and the EK-60F subscore for surprise (r = −0.3, *p* = 0.03). The HPT at the left little finger inversely correlated with the EK-60F subscore for fear (r = −0.39, *p* = 0.002). In alexithymic patients, the VDT at the left little finger inversely correlated with the RME (r = −0.52, *p* = 0.02) and the left CDT at the dorsum and the index correlated with the EK-60F subscore for surprise (r = 0.57, *p* = 0.01 and r = 0.54, *p* = 0.01, respectively). In non-alexithymic patients, the HPT at the left little finger is inversely correlated with the EK-60F subscore for fear (r = −0.50, *p* = 0.002).

### 4.6. Sensory Phenotype and Emotion Processing Characteristics

The results of the EK-60F and RME in patients grouped according to the presence and type of sensory loss (L0, L1, L2, L3) and allodynia (G0, G1, G2, and G3) are summarized in Table 4 and Table 5. Patients with L3 had a significantly lower RME than patients with L0, L1, and L2. Patients with G2 had a lower RME than patients with G0 and G1. Patients with G2 had a lower EK-60F overall score than patients with G1 and a lower subscore for surprise than patients with G0 and G1. Patients with G3 had a higher EK-60F subscore for fear than patients with G0 and G2. L0 was more common in non-alexithymic than in moderate-high alexithymic patients (12 of 37 vs. 2 of 25, *p* = 0.02).

### 4.7. General Linear Model Analysis

The following characteristics were included in the univariate analysis to assess their relationship with the QST parameters: alexithymia group, EK-60F overall score, RME, GHQ-12 score, and NRS (see Table 6). Univariate analysis showed an effect of the alexithymia group and the RME on the VDT z-score of the left index finger and of the RME on the VDT z-score of the left little finger. In the multivariate analysis, only the effect of the alexithymia group on the left index finger VDT z-score remained.

The univariate analysis revealed an effect of the EK-60F on the RME (β = 476.72, F = 32.43, *p* < 0.0001). There was no effect of years of education on the QST parameters.

## 5. Discussion

In this study, we investigated all three levels of analysis that characterize emotion processing according to the existing literature [6,11,12], i.e., emotion recognition, emotion representation, and emotion regulation, in patients with PNP. To this end, we used a series of standardized tests and the QST, i.e., a set of psychophysical tests used to assess somatosensory function.

The GLM analysis confirms the relationship between alexithymia and VDT z-scores at the left index finger described in a previous study [7]. In addition, the GLM analysis shows that the VDT z-scores at the little finger also correlates with RME, demonstrating the relationship between vibration perception at the left hand and both emotion regulation and emotion representation in patients with neuropathic pain. The striking association of vibration perception on the left hand with emotion processing is consistent with the known role of the right hemisphere in this critical skill [42,43].

About the relationship between the VDT z-scores of the QST and both the RME and EK-60F results, it is also important to note that it has been hypothesized that the skin and its somatosensory afferent projections to the somatosensory cortex make a crucial contribution to the experience of emotions [23]. Consistent with this, activation of the primary somatosensory cortex during interpersonal touch is modulated by the facial expression and gender of the touching person, as demonstrated by fMRI [44] and somatosensory evoked potential data [45]. The results reported here provide further support for this hypothesis at the psychophysiological level of analysis.

In line with previous studies [6,46,47], we found reduced performance on the overall EK-60F in alexithymic patients. In these patients, left CDT z-scores also correlated with the EK-60F subscore for surprise, which is a predictor of alexithymia [47]. In contrast, in non-alexithymic patients, sensitivity to heat pain is inversely related to the EK-60F subscore for fear. In non-alexithymic, but not in highly alexithymic subjects, emotion recognition of fear increases the accuracy of touch detection in the Visual Remapping of Touch paradigm, while this is not the case for emotion recognition of happiness [48]. This observation is consistent with the relationship we found between different sensory modalities (CDT vs. HPT) and different EK-60F subscores (surprise vs. fear) in alexithymic versus non-alexithymic patients.

In our previous report [7] and the present study, we found no association between pain intensity assessed with the NRS procedure and both disability assessed with the GHQ-12 and alexithymia. Furthermore, we found no significant association between the RME or EK-60F and pain intensity in the present study. These results are consistent with the observation that in patients with neuropathic pain, repetitive transcranial magnetic stimulation of the motor cortex reduced the intensity of perceived pain but did not alter the affective dimension of pain [49]. Similar results were found in patients with fibromyalgia [50]. These observations support the concept that the perception of pain and the “suffering” caused by the pain itself operate along different pathways and that “suffering” plays an important role in the disability associated with pain [51].

### Limitations of the Study

In the current study, some limitations should be considered when generalizing the results. Firstly, we used self-report instruments to assess psychological variables, quality of health, and social support. In addition to these instruments, structured interviews should be used. Secondly, due to the cross-sectional design of this study, it is not possible to make definitive statements about the causal relationship between the variables analyzed.

## 6. Conclusions

In this study, we have shown that patients with PNP exhibit impaired emotion recognition and emotion representation and that these abnormalities are more pronounced in patients with moderate/high alexithymia. We also show that emotion recognition and emotion representation correlate with the impairment of vibration perception on the left hand, but not with pain intensity. Together with the results of our previous study [7], the present data suggest abnormalities in emotion processing at both intrapersonal and interpersonal levels and a link between these abnormalities and somatosensory perception, particularly vibration perception.

Overall, the results here reported contribute to the understanding of the emotion processing profile of patients with PNP. The impairments reported here highlight the importance of appropriately assessing emotion processing abilities in clinical practice. In this way, clinicians may even be able to tailor treatment to the needs of each patient.

## Figures and Tables

**Table 1:** Demographic and clinical characteristics of patients and controls.

	All Patients	Moderate-High Alexithymia	No Alexithymia	Healthy Control
Age	52.7 ± 13.6	51.9 ± 12.2	53.2 ± 14.6	48.3 ± 15.5
Male/Female	24/38	13/12	11/26	18/30
Years of education	11.3 ± 3.5 ^a^	11.1 ± 3.1 ^a^	11.4 ± 3.7 ^a^	14.3 ± 4.7
Primary school diploma	5	0	5	1
Secondary school diploma	18	11	7	7
High school diploma	8	4	4	2
Graduation	31	10	21	38
NRS	6.2 ± 2.4	6.6 ± 2.2	6.1 ± 2.5	-
DN4	5.4 ± 2.2	5.6 ± 2.3	5.5 ± 2.3	-
CTS	34	13	21	-
PBHS	13	5	8	-
ONP	15	7	8	-

CTS = carpal tunnel syndrome; DN4 = Douleur Neuropathique 4-question questionnaire; NRS = 11-point numerical rating scale; ONP = other neuropathic pain (brachial plexopathy, cervical painful radiculopathy, ulnar nerve compression at elbow); PBHS = post-burn hypertrophic scars. ^a^ *p* < 0.01 versus healthy controls.

**Table 2:** Summary of CDT, HPT, and VDT z-scores at different sites in moderate-high alexithymic and non-alexithymic patients.

		Moderate-High Alexithymia	No Alexithymia
CDT			
Dorsum	R	−3.5 ± 6.1	−6.3 ± 20.9
	L	−95.3 ± 271.7	−44.9 ± 182.8
Index	R	−2.6 ± 4.1	−1.3 ± 3.8
	L	−28.3 ± 82.0 ^a^	−18.3 ± 69.0
Little finger	R	−6.5 ± 1.3	−0.9 ± 3.0
	L	−22.7 ± 51.0	−11.5 ± 35.7
HPT			
Dorsum	R	0.20 ± 0.91 ^b^	−0.21 ± 1.1
	L	0.14 ± 1.1	−0.52 ± 1.12
Index	R	0.23 ± 0.67	−0.24 ± 1.07
	L	0.21 ± 0.84	−1.05 ± 4.66
Little finger	R	−0.03 ± 6.95	−0.24 ± 1.0
	L	−0.01 ± 1.13	−0.36 ± 1.06
VDT			
Index	R	−0.94 ± 1.18	0.62 ± 1.16
	L	2.34 ± 2.44 ^a^	−0.31 ± 1.41
Little finger	R	1.08 ± 1.2	0.67 ± 1.04
	L	1.51 ± 2.18	0.7 ± 1.02

CDT = cold pain threshold; HPT = heat pain threshold; L = left; R = right; VDT = vibration detection threshold. ^a^ *p* < 0.01 versus no-alexithymia; ^b^ *p* < 0.05 versus no-alexithymia.

**Table 3:** Summary of Ekman 60-Faces Test and Reading the Mind in the Eyes Test according to presence and severity of alexithymia.

Test	All Patients	Moderate-High Alexithymia	No-Alexithymia	Healthy Controls
Ekman 60-Faces				
Overall score	46.9 ± 5.7 ^b^	46.1 ± 4.6 ^a,c^	47.6 ± 6.4	49.1 ± 5.1
Happiness	9.7 ± 0.7	9.8 ± 0.5	9.7 ± 0.8	9.8 ± 0.3
Surprise	9 ± 1.6	8.9 ± 1.3	9 ± 1.8	9.2 ± 0.9
Anger	7.1 ± 1.6	6.7 ± 1.5 ^a,d^	7.4 ± 1.5	7.6 ± 1.8
Disgust	8.5 ± 1.5	8.8 ± 1.3	8.3 ± 1.7	8.9 ± 1.2
Sadness	7.3 ± 1.6	6.7 ± 1.7 ^d^	7.7 ± 1.3	7.5 ± 1.6
Fear	5.3 ± 2.4	5.1 ± 2.2	5.4 ± 2.4	6.1 ± 2.4
RME	24 ± 6.1	22.6 ± 3.7 ^a^	24.8 ± 7.2	25.2 ± 4.3
GHQ-12	3.2 ± 4.6 ^a^	4.1 ± 4.6 ^a^	2.7 ± 4.7	1.3 ± 2

GHQ-12 = General Health Questionnaire; RME = Reading the Mind in the Eyes Test. ^a^ *p* < 0.01 versus healthy controls; ^b^ *p* < 0.05 versus healthy controls; ^c^ *p* < 0.01 versus non-alexithymic patients; ^d^ *p* < 0.05 versus non-alexithymic patients.

**Table 4:** Summary of Ekman 60-Faces Test, Empathy quotient, and Reading the Mind in the Eyes Test according to presence and severity of sensory loss.

Test	L0	L1	L2	L3
Ekman 60-Faces				
Overall score	48.4 ± 5.1	46.5 ± 4.6	47.2 ± 3.7	46.3 ± 7.6
Happiness	9.6 ± 0.8	9.8 ± 0.6	9.8 ± 0.4	9.8 ± 0.8
Surprise	9.6 ± 0.6	9.2 ± 1.0	9.1 ± 0.8	8.4 ± 2.3
Anger	7.1 ± 1.6	6.7 ± 1.4	6.7 ± 1.4	7.4 ± 1.7
Disgust	8.5 ± 1.5	8.8 ± 1.1	8.4 ± 0.9	8.4 ± 2.0
Sadness	7.4 ± 1.5	7.0 ± 1.9	7.9 ± 0.8	7.1 ± 1.7
Fear	6.4 ± 2.0	4.9 ± 2.2	5.1 ± 2.6	4.8 ± 2.5
RME	25.5 ± 4.0 ^a^	25.0 ± 4.6 ^a^	25.4 ± 10.4 ^a^	21.6 ± 4.5
GHQ-12	1.7 ± 2.0	2.8 ± 4.0	5.2 ± 7.4	3.4 ± 4.4

GHQ-12 = General Health Questionnaire; L0 = no loss of thermal/pain and vibration/touch; L1 = loss of thermal/pain; L2 = loss of vibration or touch sense; L3 = combined loss of thermal/pain and vibration/touch; RME = Reading the Mind in the Eyes Test. ^a^ *p* < 0.05 versus L3.

**Table 5:** Summary of Ekman 60-Faces Test and Reading the Mind in the Eyes Test according to presence and severity of allodynia.

Test	G0	G1	G2	G3
Ekman 60-Faces				
Overall score	46.9 ± 5.3	50.0 ± 4.7 ^a^	43.9 ± 7.2	51 ± 1.0
Happiness	9.7 ± 0.6	10 ± 0.1	9.5 ± 0.1	9.3 ± 0.6
Surprise	9.08 ± 1.7 ^a^	9.6 ± 0.7 ^a^	8.3 ± 1.7	9.0 ± 1.0
Anger	7.3 ± 1.2	7.1 ± 1.7	6.1 ± 2.1	7.6 ± 2.3
Disgust	8.4 ± 1.4	8.6 ± 1.5	8.5 ± 2.1	9.3 ± 0.6
Sadness	7.3 ± 1.6	7.9 ± 1.1	7 ± 1.7	7.6 ± 2.3
Fear	5.1 ± 2.0 ^b^	6.7 ± 2.3 ^a^	4.0 ± 2.8 ^b^	8 ± 0.0
RME	24 ± 4.5 ^a^	26.4 ± 4.1 ^a^	22.1 ± 11.1	24± 3.6
GHQ-12	2.8 ± 3.75	1.25 ± 2.18 ^a^	6.27 ± 7.5	2.66 ± 3.05

G0 = no allodynia; G1 = allodynia for heat; G2 = allodynia for mechanical stimuli; G3 = combined allodynia for heat and mechanical stimuli; GHQ-12 = General Health Questionnaire; RME = Reading the Mind in the Eyes Test. ^a^ *p* < 0.05 vs. G2; ^b^ *p* < 0.05 vs. G3.

**Table 6:** Summary of General Linear Model analysis.

	β	F	*p*
Univariate analysis			
Left index finger VDT z-score			
Ekman 60-Faces	3.77	0.87	0.32
RME	12.83	4.46	0.04
Alexithymia group	49.79	13.61	0.0006
GHQ-12	5.29	1.2	0.279
NRS	2.17	0.47	0.497
Left little finger VDT z-score			
Ekman 60-Faces	2.76	1.26	0.267
RME	9,76	7.44	0.009
Alexithymia group	9.31	3.54	0.065
GHQ-12	3.13	1.1	0.29
NRS	1.72	0.58	0.45
Multivariate analysis			
Left index VDT z-score			
RME	8.65	3.63	0.06
Alexithymia group	11.24	4.71	0.03

GHQ-12 = General Health Questionnaire; NRS = numeric rating scale; RME = Reading the Mind in the Eyes; VDT = vibration detection threshold.

## Data Availability

The data sets used and/or analyzed in the current study are available from the first author upon reasonable request.

## Supplementary Materials

The following supporting information can be downloaded at: https://www.mdpi.com/article/10.3390/medsci12020027/s1, Table S1: Summary of motor and sensitive nerve conduction study (NCS) results; Table S2: Summary of CDT, HPT, and VDT (log and ln transformed data) at different sites in moderate-high and non-alexithymic patients.
