# Supplementary material for: Emotion Processing in Peripheral Neuropathic Pain: An Observational Study
Source: Med Sci (Basel). 2024 May 17;12(2):27. doi: 10.3390/medsci12020027 (PMC11130802; doi:10.3390/medsci12020027)
Supplement: Supplementary file 1 [file medsci-12-00027-s001.zip › medsci-2949614-supplementary.pdf]

Emotion Processing in Peripheral Neuropathic Pain: An Observational Study

Gianluca Isoardo, Mauro Adenzato, Stefano Ciullo, Elena Fontana, Ilaria Stura, Giuseppe Migliaretti, Paolo Titolo, Enrico Matteoni, Andrea Calvo, Federica Laino, Francesca Palumbo and Rita B. Ardito

Table S1. Summary of motor and sensitive nerve conduction studies (NCS) results.

	MCV (m/s)		SCV (m/s)		CMAP amplitude (mV)		SAP amplitude (mV)		Latency (ms)	
	Right	Left	Right	Left	Right	Left	Right	Left	Right	Left
					Median					
All patients	52.7 ± 4.7[b]	52.9 ± 5.2[b]	47.8 ± 8.8[a]	47.8 ± 9.3[a]	9.2 ± 4.1	8.8 ± 4.9	24.7 ± 16.6[a]	28.6 ± 20.4[a]	4.2 ± 1.1[a]	4 ± 1[a]
TAS-20 ≥ 52	52.6 ± 4.6[b]	51.2 ± 5.9[b]	47.8 ± 9.5[b]	47.3 ± 10.5[a]	9.3 ± 4.4	7.1 ± 5.1	21.2 ± 14.2[b]	19.6 ± 15.4[a]	4.1 ± 1.1[a]	4.2 ± 1.1[a]
TAS-20 < 52	52.8 ± 4.9[b]	54.1 ± 4.4[b]	47.8 ± 8.5[a]	48.3 ± 8.6[a]	9.0 ± 4.1	10.2 ± 4.3	27.1 ± 17.8[b]	35.1 ± 22.9[b]	4.3 ± 1.1[a]	3.9 ± 1.0[a]
Healthy controls	57.9 ± 6.3	59.6 ± 5	59.7 ± 7.8	62.2 ± 7.6	9.6 ± 4	10.4 ± 5.9	49.7 ± 21.2	57.6 ± 21.9	3.2 ± 0.3	3.1 ± 0.4
					Ulnar					
All patients	58.6 ± 6.9	59.2 ± 7	56.7 ± 7.2	56.9 ± 7.7	8.1 ± 3.1	7 ± 3.4	28.9 ± 16.8[a]	32.1 ± 18.6[b]	2.6 ± 0.5	2.7 ± 0.6
TAS-20 ≥ 52	58.4 ± 6.8	60.0 ± 5.2	56.5 ± 5.8	54.9 ± 6.6	8.3 ± 4.0	6.4 ± 4.3	22.8 ± 12.9[b]	23.2 ± 15.4[b]	2.8 ± 0.6	2.7 ± 0.5
TAS-20 < 52	58.8 ± 7.2	58.8 ± 8.2	56.8 ± 8.0	58.4 ± 8.1	7.8 ± 2.8	7.5 ± 2.6	32.8 ± 18.0[b,d]	38.9 ± 18.2[c,d]	2.5 ± 0.3	2.7 ± 0.7
Healthy controls	59 ± 6.5	58 ± 6.2	58,5 ± 6.8	58.3 ± 6.8	9.9 ± 4.1	7.4 ± 3.8	52.4 ± 22.3	52 ± 22.8	2.7 ± 0.3	3 ± 0.5

CMAP = Compound Muscle Action Potential; m = meters; MCV = Motor Conduction Velocity; ms = milliseconds; mV = millivolts; s = seconds; SAP = Sensor Action Potential; SCV = Sensor Conduction Velocity.[a] $p < 0.0001$ versus healthy controls; [b] $p < 0.01$ versus healthy controls; [c] $p < 0.05$ versus healthy controls; [d] $p < 0.05$ versus non-alexithymic patients.

Table S2. Summary of CDT, HPT and VDT (log and ln transformed data) at different sites in moderate-high and non-alexithymic patients.

		All patients	Moderate-High Alexithymia	No-alexithymia	Healthy controls
CDT					
Dorsum	R	1.482 ± 0.056	1.488 ± 0.024	1.478 ± 0.070	1.500 ± 0.003
	L	1.303 ± 0.633 [a]	1.232 ± 0.765 [a]	1.363 ± 0.52 [a]	1.500 ± 0.002
Index	R	1.470 ± 0.051 [a]	1.459 ± 0.054 [a]	1.477 ± 0.049	1.493 ± 0.013
	L	1.297 ± 0.656 [a]	1.244 ± 0.727 [a,c]	1.334 ± 0.612	1.496 ± 0.003
Little finger	R	1.467 ± 0.051	1.473 ± 0.026	1.464 ± 0.061	1.483 ± 0.020
	L	1.196 ± 0.768 [a]	1.076 ± 0.924 [a]	1.277 ± 0.646 [a]	1.487 ± 0.018
HPT					
Dorsum	R	1.634 ± 0.041	1.640 ± 0.037	1.630 ± 0.044	1.639 ± 0.040
	L	1.632 ± 0.042	1.646 ± 0.040	1.621 ± 0.041	1.640 ± 0.637
Index	R	1.660 ± 0.034	1.608 ± 0.028	1.652 ± 0.036	1.652 ± 0.032
	L	1.635 ± 0.128	1.652 ± 0.029	1.617 ± 0.162	1.649 ± 0.034
Little finger	R	1.653 ± 0.034	1.657 ± 0.033	1.650 ± 0.034	1.658 ± 0.035
	L	1.654 ± 0.037	1.668 ± 0.023	1.649 ± 0.036	1.661 ± 0.034
VDT					

Index	R	-0.241 ± 0.942 [a]	-0.087 ± 0.950 [a]	-0.348 ± 0.934 [b]	-0.848 ± 0.805
	L	-0.109 ± 1.665 [a]	0.808 ± 1.887 [a, c]	-0.761 ± 0.94 [a]	-1.003 ± 0.773
Little finger	R	-0.194 ± 0.918 [a]	-0.153 ± 0.990 [a]	-0.319 ± 0.864	-0.876 ± 0.824
	L	-0.040 ± 1.481 [a]	0.406 ± 1.965 [a]	-0.357 ± 0.918 [a]	-0.953 ± 0.859

CDT = Cold Pain Threshold; HPT = Heat Pain Threshold; L = Left; R = Right; VDT = Vibration Detection Threshold.[a] $p < 0.01$ vs healthy controls; [b] $p < 0.05$ vs healthy controls; [c] $p < 0.01$ vs non-alexithymic patients.
